# A prospective study on the efficacy of patient simulation in heart and lung auscultation

**DOI:** 10.1186/s12909-019-1708-6

**Published:** 2019-07-23

**Authors:** Stella Bernardi, Fabiola Giudici, Maria Fontana Leone, Giulia Zuolo, Stefano Furlotti, Renzo Carretta, Bruno Fabris

**Affiliations:** Department of Medical Sciences, Università degli Studi di Trieste, Cattinara Teaching Hospital, Strada di Fiume 447, 34100 Trieste, Italy

**Keywords:** Patient simulation, Patient simulators, Heart auscultation, Lung auscultation, Medical education, Clinical education, Medical semiotics

## Abstract

**Background:**

The use of simulation technology for skill training and assessment in medical education has progressively increased over the last decade. Nevertheless, the teaching efficacy of most technologies remains to be fully determined. The aim of this prospective study was to evaluate if a short individual training on a patient simulator could improve heart and lung auscultation skills in undergraduate students.

**Methods:**

A group of fifth-year medical school students, who had trained on a patient simulator in their third year (EXP, *n* = 55), was compared to a group of fifth-year medical school students who had not previously trained on it (CNT, *n* = 49). Students were recruited on a voluntary basis. Students were evaluated in terms of their ability to correctly identify three heart (II sound wide split, mitral regurgitation, aortic stenosis) and five lung sounds (coarse crackles, fine crackles, pleural rubs, rhonchi, wheezes), which were reproduced in a random order on the Kyoto-Kagaku patient simulator.

**Results:**

Exposure to patient simulator significantly improved heart auscultation skills, as mitral regurgitation was correctly recognized by 89.7% of EXP students as compared to 71.4% of CNT students (*p* = 0.02). In addition, a significantly greater percentage of EXP students correctly graphed all the heart diagnoses as compared to CNT students. There were no differences between the groups in lung auscultation.

**Conclusions:**

This study demonstrates that training medical students with a patient simulator, individually for one hour, significantly ameliorated their heart auscultation skills. Our data suggests that patient simulation might be useful for learning auscultation skills, especially when it is combined with graphic sound display.

## Background

Cardiopulmonary auscultation is a pivotal part of any physical examination [1]. It allows for the early detection of critical signs, it helps in the selection of more complex and expensive tests, and – by requiring the physical contact between physician and patient, it might have an immediate therapeutic value [2]. Cardiopulmonary auscultation has always been classically taught at the patient bedside [3]. Nevertheless, teaching at the patient bedside is limited by a relatively large student-to-patient ratio, the heterogeneity/variability of clinical presentations, as well as by the inconvenience of repeated physical examinations to patients with advanced disease [4]. Moreover, there might be specific circumstances, such as sudden outbreaks of contagious diseases, which can severely limit traditional bedside teaching, as it has been interestingly reported by Lam and colleagues [5]. In addition, it has been pointed out that clinical practice does not necessarily correlate with skill in auscultation [2, 6].

Technology and high fidelity patient simulators represent new valuable instruments of instruction, skill acquisition, and assessment in medicine [7]. By contrast to bedside teaching, patient simulators are readily accessible at any time. High fidelity patient simulators can be further divided into skill-trainer, computer-enhanced, and virtual reality simulators [8]. Skill-trainer simulators offer the possibility to exercise heart and lung auscultation [9]. Not only do they offer the possibility to reproduce and compare a wide variety of conditions on demand, but also to expose every student in a class to the same specific heart or lung sound, thereby providing a standardized experience for all [8]. In addition, simulators represent a convenient, reliable, and objective method/tool for auscultation skill assessment [6]. Consistent with these characteristics, patient simulators are likely to favour not only the acquisition and evaluation of professional skills, but also a democratization of medical education.

With respect to the efficacy of patient simulators in cardiopulmonary auscultation, Ewy and colleagues have shown that the cardiology patient simulator improved the knowledge and the skills necessary to perform a cardiovascular examination [4]. Nevertheless, other authors found little evidence that students trained with a patient simulator were more able to transfer skills to real patients [10]. So, the teaching efficacy of most technologies remains to be fully determined. Based on these premises, the current prospective study aimed to evaluate if a short individual training with a patient simulator (in addition to the conventional bedside teaching) could improve heart and lung auscultation skills. For this purpose, we compared two groups of fifth-year medical school students enrolled at the University of Trieste; one group had trained with the patient simulator during their third year whereas the other had not.

## Methods

### Population

**Fig. 1:**
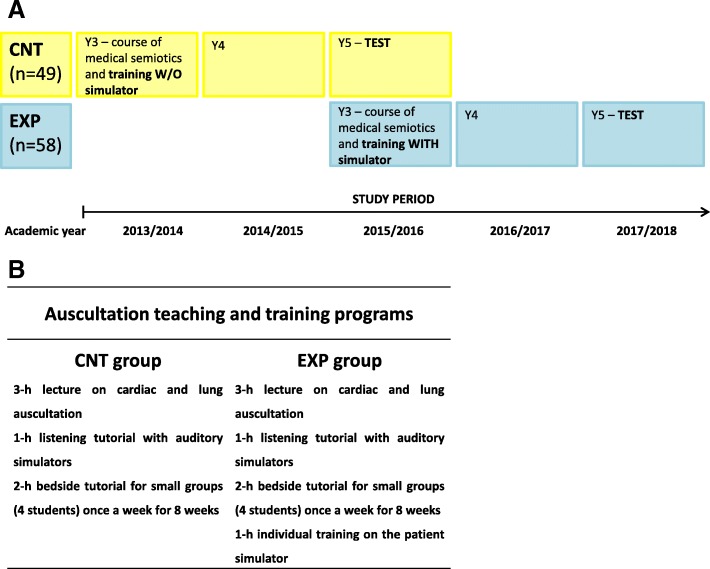
(**a**) Protocol of the study. (**b**) Auscultation teaching and training programs undertaken by the control and exposed groups, respectively, during the course of Medical Semiotics. The introduction of an individual 1-h training on the patient simulator took place in the academic year 2015/2016. CNT is for control, EXP is for exposed

This is a prospective study carried out at the Medical School of the Department of Medical Sciences of the University of Trieste between 2013 and 2018. This study was designed to evaluate the efficacy of training with a patient simulator in heart and lung auscultation. For this purpose, we compared two groups of fifth-year medical school students enrolled at the University of Trieste who had already taken the exam of medical semiotics in their third year (i.e. when students learn to perform a physical examination). Students were recruited on a voluntary basis in their fifth year and provided informed consent to participate in this study. The study was performed in accordance with the standards of the Declaration of Helsinki. The first group, also called the control group (CNT group; 49 students), included fifth-year medical students who had never trained with the patient simulator before this study. These students had taken the exam of medical semiotics in the academic year 2013/2014, and were tested in the first semester of the academic year 2015/2016. The second group, also called the exposed group (EXP group; 58 students), included fifth-year medical students who had trained individually for one hour with the patient simulator before taking the exam of medical semiotics. These students had taken the exam of medical semiotics in the academic year 2015/2016, and were tested in the first semester of the academic year 2017/2018. It should be noted that the percentage of students who volunteered was the same in both cohorts, as the CNT group consisted of 49 fifth-year students out of a cohort of 141 students (34%), while the EXP group consisted of 58 students out of a cohort of 167 students (34%). It is likely that the students who volunteered were the students most motivated in their respective groups. Therefore, this recruitment modality might have helped to increase the student level of care during the test. The protocol for this study and the training programs undertaken by the two groups are reported in Fig. 1a-b.

### Auscultation training and testing

Training. Figure 1b summarizes the auscultation teaching programs undertaken by the two groups. Lectures and tutorials were given by the same instructor to both classes (i.e. in the academic year 2013/2014 and 2015/2016), which might have helped to reduce the biases due to different teaching methods. Each auscultation teaching program was part of the students’ mandatory course of medical semiotics, which was scheduled in their respective third year. Briefly, the CNT group had to attend a three-hour lecture on cardiac and lung auscultation, a one-hour listening tutorial with auditory simulators, as well as a two-hour bedside tutorial (clerkship) in small subgroups once a week for eight weeks. During the one-hour listening tutorial with auditory simulators the following sounds/murmus were played: aortic stenosis, mitral regurgitation, aortic regurgitation, and mitral stenosis, III and IV sounds, as well as wheezes, ronchi, coarse and fine crackles, and pleural rubs. Two years later, the EXP group attended the same lectures and tutorials of the CNT group, which should have provided the basis for equal basal auscultation skills to both groups. It must be noted that, be it the CNT or the EXP group, during these lectures and tutorials (Fig. 1b), students were taught how to recognize different heart conditions through the graphical representation of heart sounds and murmurs, which would guide them to the correct diagnosis. In particular, they were taught to recognize if a sound/murmur was systolic or diastolic, in addition to the recognition of its location (and irradiation), intensity, duration, and shape. At the end of any cardiac auscultation they had to provide a graphical representation of what they had heard and the respective diagnosis. As for lung auscultation, they were taught to recognize the main lung sounds by their broad characteristics [11] and by matching them to the correct diagnoses.

In addition to these lectures and tutorials (Fig. 1b), the EXP group undertook an individual one-hour training with the Kyoto-Kagaku patient simulator (Cardiology patient simulator “K Plus” training system, Model #11257–159, Kyoto Kagaku Co. Ltd., Kyoto, Japan). During this training with the patient simulator, EXP students had to complete a paper, where they had to identify and graphically represent three consecutive heart sounds/murmurs, and to identify five consecutive lung sounds. These were the II sound wide split, mitral regurgitation, and aortic stenosis for the heart, which were chosen based on valvular heart disease epidemiology [12] as well as on their characteristics which we believed were appropriate as a starting point to train beginners. Coarse crackles, fine crackles, pleural rubs, rhonchi, and wheezes were chosen for the lung. All these sounds and murmurs were played in a random order. Students had 5 min to listen to each heart sound/murmur and 3 min to graphically represent it. Then, they had 3 min to listen to each lung sound and to match it to the respective diagnosis. All these sounds were reproduced in random order.

**Fig. 2:**
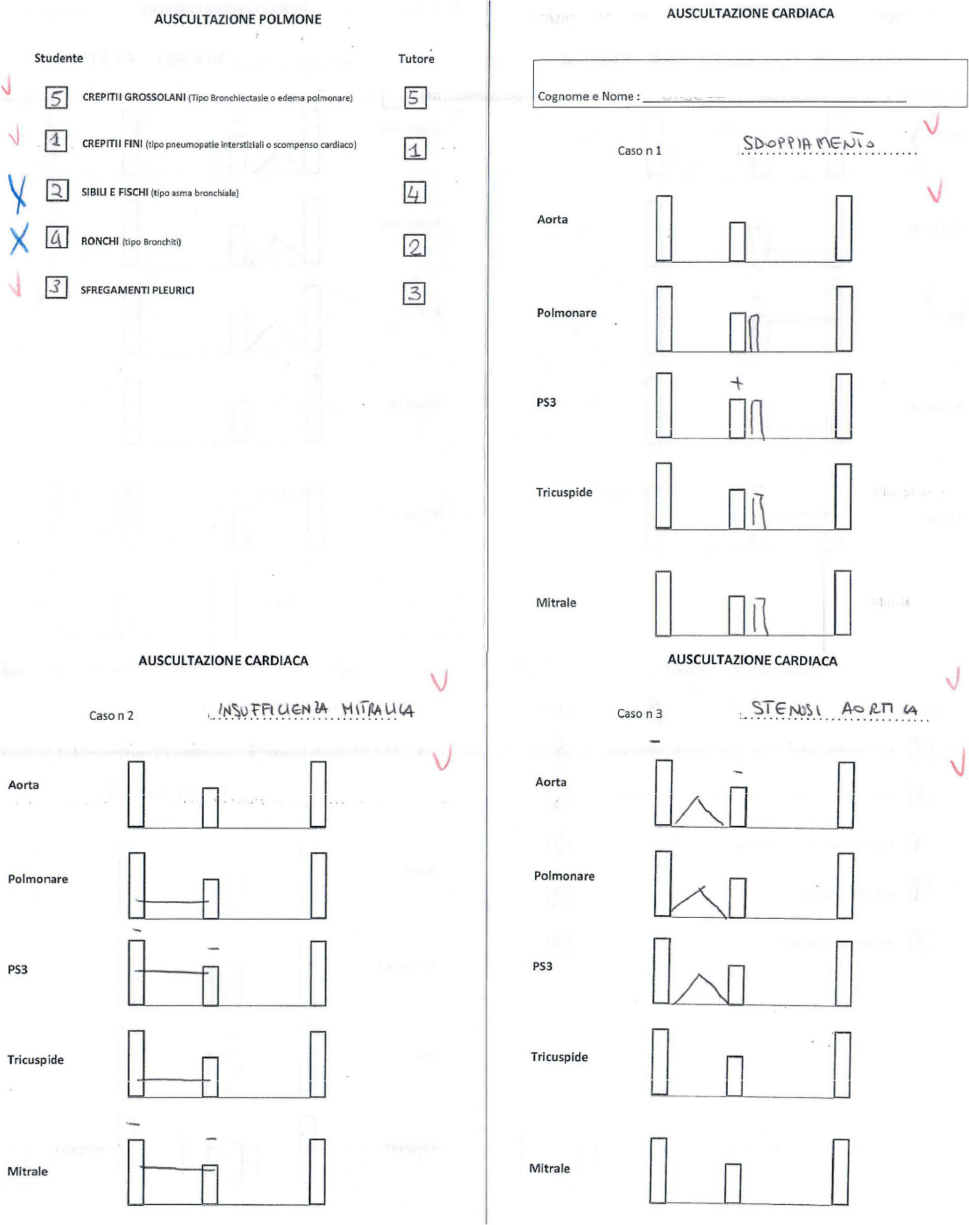
Example of the paper that students had to complete. This is a paper that has been completed by one of the students of the EXP group in their fifth year. Students had to identify and graphically represent three consecutive heart sounds/murmurs and to identify five consecutive lung sounds. Red ticks indicate the diagnoses and graphic representations that were judged correct. Blue crosses indicate the diagnoses and/or graphic representations that were judged incorrect. Reading it clockwise, *auscultazione polmone* is for pulmonary auscultation, *studente* is for student, *tutore* is for tutor, *crepitti grossolani* is for coarse crackles, *crepitii fini* is for fine crackles, *sibili e fischi* is for wheezes, *ronchi* is for ronchi, *sfregamenti pleurici* is for pleural rubs. *Auscultazione cardiaca* is for cardiac auscultation, *cognome e nome* is for name and surname, *caso* is for case, *sdoppiamento* is for II sound split, *aorta* is for aortic area, *polmonare* is for pulmonic area, *PS3* is for Erb’s point, *tricuspidale* is for tricuspid area, *mitrale* is for mitral area, *insufficienza mitralica* is for mitral regurgitation, and *stenosi aortica* is for aortic stenosis

Testing. Two years later, fifth-year CNT and EXP students, who were recruited on a voluntary basis, were tested with the Kyoto-Kagaku patient simulator on the same heart and lung sounds (II heart sound wide split, mitral regurgitation, aortic stenosis, coarse crackles, fine crackles, pleural rub, rhonchi, and wheezes), which were reproduced in random order. The students did not know the murmurs and sounds that we would play. All of them had to complete the same paper (Fig. 2) that we used for the third-year training of the EXP students. It must be noted that for lung auscultation, students were asked to match the sound with a diagnosis, while for heart auscultation, they were asked to provide both the graphical representation of what they had heard and the corresponding diagnosis. In general, graphical representation was judged correct when the murmur/split was placed in the correct pause/order and in the right location, and was represented with the right intensity. All responses were analyzed by three independent instructors. The rationale underlying the difference in the teaching/testing method of heart and lung auscultation comes from the tradition of teaching heart auscultation with the support of graphic sound display (i.e. phonocardiography) [13, 14].

### Statistics

Results were analyzed with the software R (version 3.3.2; 2016). A *p*-value < 0.05 was considered statistically significant. The groups (CNT vs EXP) were compared with the Chi-Square Test of Independence, in order to assess if there were any differences in the distribution of correct and incorrect answers between the groups. The McNemar’s test was used to compare the performances of the same EXP students between their third and fifth year.

## Results

### Training with the patient simulator significantly improved student heart auscultation skills

When the students had to identify heart sounds and murmurs, aortic stenosis was correctly diagnosed by 77.6% of CNT students and by 84.5% of EXP students (*p* = 0.36) (Table 1). Nevertheless, the percentage of students who correctly graphed the aortic stenosis murmur was 71.4% in the CNT group, while it remained 87.9% in the EXP group (*p* = 0.03 vs. CNT group) (Table 1). Second, mitral regurgitation was correctly diagnosed by 71.4% of CNT students and by 89.7% of EXP students (*p* = 0.02) (Table 1). The difference between the groups remained significant when they had to graphically represent the mitral regurgitation murmur, with only 67.3% of CNT students correctly representing it, as compared to 86.2% of EXP students (*p* = 0.02) (Table 1). Third, the splitting of the second sound was correctly diagnosed by 79.6% of CNT students and by 89.7% of EXP students (*p* = 0.15) (Table 1). Both groups encountered difficulty in providing the correct graphical representation of the splitting, though this was more evident in the CNT group, where only 30.4% of the students correctly graphed the splitting, as compared to 55.2% of the EXP students (*p* = 0.01) (Table 1).

**Table 1:** Ability of making the correct heart and lung diagnoses and correctly representing the heart sounds/murmurs

HEART AUSCULTATION				
	CNT (=49)		EXP (=58)	*p* value
AORTIC STENOSIS (diagnosis)	*Correct*	**38**	**49**	n.s.
	*Incorrect*	11	9	
AORTIC STENOSIS (representation)	*Correct*	**35**	**51**	***p*** **= 0.03**
	*Incorrect*	14	7	
MITRAL REGURGITATION (diagnosis)	*Correct*	**35**	**52**	***p*** **= 0.02**
	*Incorrect*	14	6	
MIRAL REGURGITATION (representation)	*Correct*	**33**	**50**	***p*** **= 0.02**
	*Incorrect*	16	8	
II SOUND WIDE SPLIT (diagnosis)	*Correct*	**39**	**52**	n.s
	*Incorrect*	10	6	
II SOUND WIDE SPLIT (representation)	*Correct*	**15**	**32**	***p*** **= 0.01**
	*Incorrect*	34	26	
LUNG AUSCULTATION				
	CNT (=48)		EXP (=58)	*p value*
WHEEZES (diagnosis)	Correct	**44**	**53**	n.s
	Incorrect	4	5	
RONCHI (diagnosis)	Correct	**36**	**43**	n.s
	Incorrect	12	15	
COARSE CRACKLES (diagnosis)	Correct	**32**	**41**	n.s
	Incorrect	16	17	
FINE CRACKLES (diagnosis)	Correct	**28**	**43**	n.s
	Incorrect	20	15	
PLEURAL RUBS (diagnosis)	Correct	**30**	**37**	n.s
	Incorrect	18	21	

The entries in boldface are the responses that were judged correct or that were significantly different between the groups

### Training with the patient simulator did not significantly improve student lung auscultation skills

When students listened to the chest, they needed to identify the following five lung sounds: wheezes, rhonchi, fine crackles, coarse crackles, and pleural rubs (Table 1). Wheezes were correctly recognized by 91.7% of CNT students and by 91.4% of EXP students (*p* = 0.96). Rhonchi were correctly recognized by 75% of CNT students and by 74.1% of EXP students (*p* = 0.92). As for nonmusical sounds, the EXP group showed only a tendency towards better performance, which, however, was not statistically different from the CNT group. Fine crackles were correctly identified by 58.3% of CNT students and by 63% of EXP students (*p* = 0.08). Coarse crackles were correctly identified by 66.7% of CNT students and by 70.7% of EXP students (*p* = 0.66). Pleural rubs were correctly identified by 62.5% of CNT students and by 63.8% of EXP students (*p* = 0.89).

### Students trained with the patient simulator maintain their skills during time

It must be noted that when we compared the performances of the same EXP students between year three and year five, there were no changes in the heart auscultation results, whereas they significantly improved over time in lung auscultation. In particular, EXP students recognized more often fine crackles (*p* = 0.04) and pleural rubs (*p* = 0.003), while there were no differences in either coarse crackles (*p* = 0.14) or wheezes and rhonchi recognition (*p* = 0.71 and 0.24 respectively). When examining the third-year responses, EXP students performed significantly better in heart auscultation than in lung auscultation. In heart auscultation, 91% of the students either correctly identified all the sounds/murmurs or at least the majority of them (2 out of 3). In lung auscultation, only 73% of the students correctly identified either all sounds or the majority of them (3–4 out of 5; *p* = 0.03 vs heart auscultation).

## Discussion

In this study, we show for the first time that a short individual training with a patient simulator significantly improved heart auscultation skills. In particular, a greater percentage of students who trained on the patient simulator correctly recognized mitral regurgitation, as compared to those students who did not train with the simulator. Moreover, a greater percentage of the students who trained with the simulator correctly represented all the heart sounds and murmurs, indicating that the introduction of this technology had a positive impact on heart auscultation skill acquisition in the long term. These findings provide relevant support to prior research that has reported an increase in the likelihood of diagnosing heart abnormalities on a real patient after exposure to simulator training on cardiac murmurs [3, 15].

By contrast, there were no significant differences in lung auscultation between the groups. This data indicates that our current use of the patient simulator for lung auscultation should be revised as it had no significant impact on the quality of learning outcomes. In particular, we hypothesize that the differential impact that the use of the patient simulator had on heart as compared to lung auscultation could be ascribed to the different teaching/learning method that was used. On one hand, in heart auscultation, students were taught to recognize the cardiac conditions through the graphical representation of cardiac sounds and murmurs, which should guide them to the correct diagnosis. Thus, they learned to disassemble and to analyze more in depth/detail what they were listening to. This method, which requires to relate murmur timing to heart sounds, is a legacy from the past, when cardiac examination was taught with the help of phonocardiography, and the visual display of sounds was used not only for purposes of teaching, but also to provide permanent medical records [13, 14]. On the other hand, in lung auscultation, students had only to match a diagnosis to what they were listening to. Our data suggests that the support of graphic sound display/representation might be beneficial to the acquisition of auscultation skills. Therefore, it is possible that an approach that would encourage the recognition of other characteristics than the quality of lung sounds (such as their timings and patterns), could improve the quality of the learning outcome also in lung auscultation.

The finding that the use of a patient simulator improves the acquisition of heart auscultation skills is consistent with the earlier observation by Ewy and colleagues that this tool can make a significant contribution to the teaching of auscultation [4]. In that work, it was demonstrated that after the clerkship, the students who trained with the patient simulator scored significantly higher on a multiple-choice test and a skill test on the patient simulator, as well as on cardiology patients, than those who received a traditional bedside training [4]. One of the explanations of the authors was that, in bedside teaching rounds, students spend less time practicing at the patients’ bedside than the scheduled time slot, for reasons such as patient unavailability, the poor physical condition of the patient for practicing skills, and missing laboratory data [4]. Moreover, students expressed positive attitudes towards the patient simulator, as it made learning a creative endeavor, and it held the attention as it was an innovative device. As compared to the work by Ewy and colleagues, here we show for the first time that training with a patient simulator improves heart auscultation skills over a longer period of time, as students were tested two years after their training [4]. On the other hand, as compared to previous works [10], including that by Ewy [4], we did not evaluate students’ performances on real patients, which is an area that needs further studies.

It is also noteworthy that we did not find any change in cardiac auscultation when we compared the performances of the same EXP students between their third and fifth year, whereas they improved in lung auscultation. Taken together, our data indicate that the skills acquired with the patient simulator (during the third-year course of medical semiotics) are maintained (and they might even improve) over time. This is in line with the study of Perlini [16] who showed that 12.1% of students had the ability to diagnose five cardiac pathologic conditions before training with the simulator, that this percentage rose to 73.1% directly after the training, and that it remained at 68.4% three years later. Based on these findings, Perlini and colleagues concluded that the improvement in auscultation skills was maintained over time [16]. Our finding that the performances of EXP students in heart auscultation did not change between their third and fifth year is also consistent with the report by Vukanovic-Criley [6], who showed that cardiac examination skills did not improve after third-year medical school (except for cardiologists), which highlights the importance of undergraduate medical education. As for lung auscultation, to our knowledge there are no studies evaluating skill retention over time. In both cases, further studies are needed to clarify what could be the best strategies for long term skill retention/improvement (i.e. from students to trainees, faculty, and practicing physicians) and what is the value of repeated/deliberate practice.

## Conclusion

In conclusion, this study demonstrates that training medical students with a patient simulator, individually for one hour, significantly ameliorated their heart auscultation skills over time. Moreover, this study suggests that patient simulation might be useful for learning auscultation skills especially if it is combined with the graphic sound display.

## Data Availability

The datasets supporting the conclusions of this articles are available from the corresponding author on reasonable request at stella.bernardi@asuits.sanita.fvg.it.

## Abbreviations

CNT: is for control students
EXP: is for students trained individually and for one hour on a patient simulator

## References

[1] Chizner MA (2008). Cardiac auscultation: rediscovering the lost art. Curr Probl Cardiol.

[2] Mangione S, Nieman LZ, Gracely E, Kaye D (1993). The teaching and practice of cardiac auscultation during internal medicine and cardiology training. A nationwide survey. Ann Intern Med.

[3] Butter J, McGaghie WC, Cohen ER, Kaye M, Wayne DB (2010). Simulation-based mastery learning improves cardiac auscultation skills in medical students. J Gen Intern Med.

[4] Ewy GA, Felner JM, Juul D, Mayer JW, Sajid AW, Waugh RA (1987). Test of a cardiology patient simulator with students in fourth-year electives. J Med Educ.

[5] Lam CS, Cheong PY, Ong BK, Ho KY (2004). Teaching cardiac auscultation without patient contact. Med Educ.

[6] Vukanovic-Criley JM, Criley S, Warde CM, Boker JR, Guevara-Matheus L, Churchill WH (2006). Competency in cardiac examination skills in medical students, trainees, physicians, and faculty: a multicenter study. Arch Intern Med.

[7] Issenberg SB, McGaghie WC, Hart IR, Mayer JW, Felner JM, Petrusa ER (1999). Simulation technology for health care professional skills training and assessment. JAMA..

[8] Scalese RJ, Obeso VT, Issenberg SB (2008). Simulation technology for skills training and competency assessment in medical education. J Gen Intern Med.

[9] Ward JJ, Wattier BA (2011). Technology for enhancing chest auscultation in clinical simulation. Respir Care.

[10] de Giovanni D, Roberts T, Norman G (2009). Relative effectiveness of high- versus low-fidelity simulation in learning heart sounds. Med Educ.

[11] Bohadana A, Izbicki G, Kraman SS (2014). Fundamentals of lung auscultation. N Engl J Med.

[12] Iung B, Vahanian A (2014). Epidemiology of acquired valvular heart disease. Can J Cardiol.

[13] Tavel ME, Brown DD, Shander D (1994). Enhanced auscultation with a new graphic display system. Arch Intern Med.

[14] Tavel ME (2006). Cardiac auscultation: a glorious past--and it does have a future!. Circulation..

[15] Fraser K, Wright B, Girard L, Tworek J, Paget M, Welikovich L (2011). Simulation training improves diagnostic performance on a real patient with similar clinical findings. Chest..

[16] Perlini S, Salinaro F, Santalucia P, Musca F (2014). Simulation-guided cardiac auscultation improves medical students' clinical skills: the Pavia pilot experience. Intern Emerg Med.

